# The Effect of Antigen Retrieval on Cells Fixed in 10% Neutral Buffered Formalin Followed by Transfer to 70% Ethanol

**DOI:** 10.1371/journal.pone.0082405

**Published:** 2013-12-17

**Authors:** Dennis Otali, Qinghua He, William E. Grizzle

**Affiliations:** 1 Department of Pathology, University of Alabama at Birmingham, Birmingham, Alabama, United States of America; 2 Department of Biology, University of Alabama at Birmingham, Birmingham, Alabama, United States of America; 3 Department of Chemical Engineering, Tuskegee University, Tuskegee, Alabama, United States of America; University of Palermo, Italy

## Abstract

Fixation in 10% neutral buffered formalin prior to transfer to 70% ethanol for one week has been shown to adequately preserve immunorecognition of PCNA, cytokeratins AE1/AE3 and EGFr. This study investigated whether 12 hrs fixation in 10% NBF plus transfer to 70% ethanol for 4 weeks would similarly preserve immunorecognition to an extent where antigen retrieval (AR) used to reverse the masking effects of fixation on some antigens would not be necessary. Two cell lines, DU145 and SKOV3 were grown on coverslips and fixed either for 684 hrs in 10% NBF or for 12 hrs in 10% NBF which was then replaced with 70% ethanol for 672 hrs. The second experiment had the same design except an additional set of cells were subjected to heat-induced AR concomitantly. PCNA, cytokeratins AE1/AE3, and EGFr (membrane and cytoplasmic) were used to evaluate the effects of immunorecognition. Fixation in 10% NBF for 12 hrs plus transfer to 70% ethanol for 672 hrs did not preserve immunorecognition of PCNA adequately in either cell lines. Cytokeratins immunoreactivity was preserved by transfer to 70% ethanol. Cytoplasmic EGFr antigens were not adversely affected by 10% NBF fixation in either cell line and transfer to 70% ethanol had limited effects. With AR, there was little recovery of PCNA immunorecognition on cells fixed in only 10% NBF, but almost complete recovery for cells transferred to 70% ethanol. For cytokeratins there was complete recovery of immunorecognition either with only 10% NBF or 12 hrs plus transfer to 70% ethanol. For EGFr, AR resulted in complete loss of immunorecognition following either treatment. This study indicated that 12 hrs of fixation in 10% NBF plus transfer to 70% ethanol for 4 weeks with AR resulted in recovery of immunorecognition for PCNA and cytokeratins, but standard methods of AR caused loss of immunorecognition of EGFr.

## Introduction

Since the mid-20^th^ century, 10% neutral buffered formalin (NBF), an aldehyde fixative which cross links specific molecules, has been predominantly used for fixation of tissues and cells in research and diagnostic pathology [1], [2]. This fixative rapidly penetrates tissues and preserves cellular organelles causing minimal distortion [3]. In addition, 10% NBF fixation enables tissues to withstand subsequent steps in tissue processing resulting in consistent staining and long term stability on storage of the paraffin blocks [1], [3], [4]; however, exposure to 10% NBF for more than 18 hrs decreases immunorecognition of some antigens [5]–[8].

Several functional groups of biological molecules react with 10% NBF through the formation of reactive hydroxymethyl groups, Schiff-bases and methylene bridges depending upon the time of exposure. The reaction of 10% NBF with primary amines (e.g. lysine) and thiols (e.g. cysteine) results in the formation of the hydroxymethyl groups [9] while its reaction with side chains of tryptophan and lysine in the formation of a Schiff-bases i.e., intermediate imine groups [10]. These two reactions occur rapidly. Upon longer exposure to 10% NBF, the hydroxymethyl and imine groups from the initial reactions react with other side chains such as glutamine and tyrosine to form methylene-bridge cross-links [10]. This is the reaction that is thought to result in loss of immunorecognition of selective antigens [5], [10]. During tissue processing, the hydrophobic environment created in tissues has been shown to interact with fixation to modify immunorecognition [7]. To circumvent effects on immunorecognition of aldehyde fixatives, non-aldehyde based fixatives such as 70% ethanol sometimes have been used alone or in combination with formaldehyde [5]. Non-aldehyde based fixatives are not used routinely due to hardening of tissues caused by removal of water by alcohols as well as due to other variable effects of ethanols on the preservation of specific antigens.

For too long the practice of transferring tissues from 10% NBF to 70% ethanol to avoid masking effects of formalin on some antigens has been anecdotal. A recent study demonstrated that immunorecognition of PCNA, cytokeratins AE1/AE3, EGF-receptor and Ki67 MIB-1 was preserved by the transfer from 10% NBF to 70% ethanol for up to one week; however, effects of long term fixation or storage of cells in ethanol was not determined [8]. In addition, the effect of antigen retrieval (AR) on immunorecognition of these antigens was not evaluated.

The current study examines the effects of long term storage of cells (e.g., up to 4 weeks) in 70% ethanol after an initial 12 hrs in 10% NBF and specifically seeks to determine which approach (AR versus transfer to 70% ethanol) better preserves or recovers immunorecognition. This study demonstrated that after 4 weeks of fixation in either condition, neither approach was optimal to preserve or recover immunorecognition for all antigens.

## Methods

### Experimental design

The study included two experiments: the first experiment was a 4 week fixation study which compared cells grown on coverslips and fixed for 684 hrs in 10% NBF with 12 hrs fixation in 10% NBF followed by transfer to ethanol for 672 hrs. The second experiment was identical but included AR i.e., 4 weeks of the two conditions of fixation with and without AR. For comparison, each of the two experiments was run with 5 min (0.083 hrs) of 10% NBF fixation representing a condition of minimal fixation. This short period of fixation was necessary to ensure that cells remained attached to the coverslips [8]. Both experiments were repeated independently three times at room temperature (RT).

Experiment 1 (Table 1) was designed to evaluate the effect on immunorecognition after 12 hrs fixation in 10% NBF followed by transfer to 70% ethanol for 4 weeks. Subsequently the question of whether AR could recover immunorecognition completely so that transfer to ethanol would not be necessary was considered. This necessitated repeating the initial experiment but including AR treatment i.e., pressure cooker (15 psi) heat treatment for 5 min in basic solution (EDTA) at pH 8.0. The pH 8.0, EDTA was selected because pH 9.0 Tris EDTA proved too harsh for the cells grown on coverslips and pH 6.0 citrate did not result in optimal staining for the antigens of interest.

**Table 1:** Experimental design.

	No AR		AR	
	10% NBF (Hours)	70% ethanol (Hours)	10% NBF (Hours)	70% ethanol (Hours)
**Experiment 1**	0.083	NT	NT	NT
	684	NT	NT	NT
	12	672	NT	NT
**Experiment 2**	0.083	NT	0.083	NT
	684	NT	684	NT
	12	672	12	672

= not tested; AR = Antigen retrieval. NT

The results from experiment 2 indicated losses of immunostaining for cytoplasmic and membrane EGFr, which was not expected. Because the pH used in experiment 2 might have caused the loss in staining for EGFr, additional cells grown on coverslips were fixed in 10% NBF for 5 min, 180 hrs or 12 hrs followed by transfer to 70% ethanol for 168 hrs to enable simultaneous staining of four AR solutions as well as no AR. The AR solutions tested were: 0.01 M citric acid pH 6.0, 0.01 M EDTA pH 8.0, Tris EDTA pH 9.0 and un-buffered distilled water (GIBCO) (pH∼5.8 before AR and ∼pH 6.8 after AR). In this ‘one week fixation-experiment’ positive control coverslips staining for cytokeratins AE1/AE3 were included for each staining run. The effects of pH 6.0 and 9.0 on immunorecognition of EGFr after one week of fixation were evaluated and loss of staining similar to experiment 2 were obtained (data not shown).

### Cell culture

The cell culture techniques, immunostaining methods, and evaluation were performed as previously described [8]. Briefly, two cell lines, DU145, a prostate cancer cell line, and SKOV3, an ovarian cancer cell line, obtained from American Type Culture Collection (ATCC) were grown on coverslips. The cell lines were maintained in RPMI 1640 and DMEM, respectively, with 10% fetal calf serum plus supplements, MEM vitamin solution (Gibco, Grand Island, NY) l-glutamine (Gibco), antibiotic-antimycotic solution of penicillin, streptomycin and amphotericin B (Gibco) in an incubator with 5% CO_2_ at 37°C. In the ‘4-week fixation’ experiment, the cells were fixed in 10% NBF at RT for either 5 min, 684 hrs or 12 hrs followed by transfer to 70% ethanol for 672 hrs at RT. Prior to immunostaining, the cells were placed in Tris buffer, pH 7.6, for 10 min, permeabilized by dehydrating through graded concentrations of ethanol, i.e., 70%, 95%, and absolute ethanol and treated with acetone for 15 seconds. The cells were then rehydrated through graded concentrations of ethanol, i.e., absolute, 95%, and 70%, before transferring to a Tris bath. This method of permeabilization was found to result in optimal staining for the antigens under study.

### Immunohistochemistry

Following permeabilization, endogenous peroxidase was quenched with 3% hydrogen peroxide and non-specific staining was blocked with 3% goat serum for 1 hr at room temperature. The three primary murine monoclonal antibodies used for immunostaining are described in Table 2. Low concentrations of each primary antibody were selected in order to increase the sensitivity of immunostaining to small changes in fixation.

**Table 2:** Clones, sources and concentrations of the antibodies used.

Antibody	Clone	Source	Concentration
PCNA	PC-10	Santa Cruz Biotechnology, Santa Cruz, CA	1 µg/ml
anti-cytokeratins	AE1/AE3	Boehringer Mannheim Corp, Indianapolis, IN	5 µg/ml
EGFr	31G7	Zymed, San Francisco, CA	3 µg/ml

The primary antibodies were diluted in 1% bovine serum albumin, 1 mM EDTA in PBS, (PBE), pH 7.6. The antibodies were incubated on cells for 1 hr at room temperature. The cells were washed in Tris buffer and incubated with multispecies biotinylated goat anti-mouse/rabbit secondary antibody (Signet, Dedham, MA) for 10 min, washed again, and incubated with HRP-conjugated streptavidin (Signet) for 5 min. For each independent experimental repeat, all immunostaining was performed concomitantly, i.e., 5 min of 10% NBF fixation was performed just prior to immunostaining. Diaminobenzidine (DAB) substrate kit (Biogenex, San Ramon, CA) was used for visualization of the antigen/antibody complex and after immunostaining cells were counterstained with Mayer's hematoxylin (Sigma-Aldrich, St. Louis, MO) for 1 minute 15 seconds. The coverslips were dehydrated through graded ethanols, cleared in xylene and mounted using Permount (Fisher Scientific).

### Antigen Retrieval

To investigate the effect of AR on 12-hr 10% NBF fixation followed by transfer to 70% ethanol, cells were grown on coverslips in two sets and exposed to either AR or no AR prior to immunostaining so that the recovery of immunorecognition could be evaluated with AR (+AR) and without AR (−AR) for each of the three conditions of fixation (5 min 10% NBF, 684 hrs 10% NBF or 12 hrs 10% NBF followed by transfer to 70% ethanol for 672 hrs). As in experiment 1, all immunostaining for these conditions were performed simultaneously (Table 1).

At the designated end points of fixation, the −AR cells were placed in a Tris bath at room temperature, while the +AR were placed in a Tris bath for 10 min, rinsed in DI water, transferred to a metal coverslip rack, and placed in a Pyrex bowl containing AR solution (0.01M EDTA, pH 8.0) at 120°C. The Pyrex bowl was transferred into a pressure cooker and the pressure was maintained at 15 psi for 5 min. Pressure was allowed to drop gradually and cells in the AR buffer cooled before proceeding with immunostaining. Both sets of cells were then permeabilized and immunostained as described above.

### Evaluation of Immunohistochemistry

Blinded evaluation was performed by a board certified diagnostic pathologist (W.E.G.). Each antibody for a specific cell line at each condition and for each of the three replicate experiments was evaluated during the same session to maximize consistency. Two parameters were evaluated: the percentage of cells staining and an immunostaining score based on the sum of the intensity from 0 (no staining) to 4 (strongest staining) multiplied by the proportion of cells staining at each intensity [7], [8], [11], [12]. Where appropriate, intracellular localization was evaluated separately, e.g., for EGFr cell membrane staining was evaluated separately from cytoplasmic staining.

For statistical analysis, two-tailed t-tests in the Statistics Toolbox of MATLAB (Mathworks) were performed to assess the differences in immunostaining between the means of different samples. Differences were considered statistically significant if *p*≤0.01. This higher level of statistical significance (compared to *p*≤0.05) was chosen to minimize identifying statistically significant but small changes in immunostaining that would not be experimentally important.

Although there were statistically significant differences between the means of the some replicate experiments at some time points, overall the differences between replicate experiments were not experimentally significant; therefore data from all three replicate experiments were combined to determine percentage of cells staining and their immunostaining score. Figures and statistical analyses of immunostaining score are presented because the immunostaining score was found to be more sensitive to changes in immunorecognition than the percentage of cells staining [7], [8], [11].

## Results

### 4 week fixation of DU145 and SKOV3 cells

**Figure 1:**
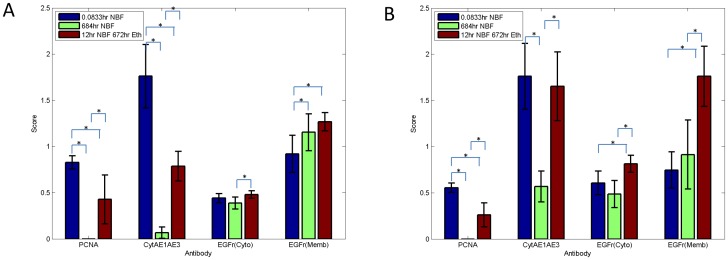
Immunostaining score of DU145 cells (panel A) and SKOV3 cells (panel B) after fixation in 10% NBF for 5 min, 684 hrs or 12 hrs plus 672 hrs in 70% ethanol and stained for PCNA, cytokeratins AE1/AE3 and EGFr. Data from are from three replicate experiments. Error bars are standard deviations (SD) with *n* = 3. * *p* value<0.01.

**Figure 2:**
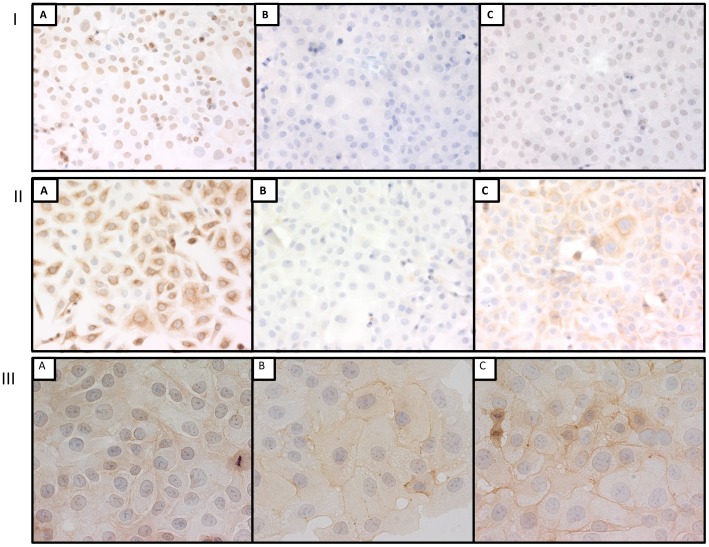
Panels I, II and III represent immunostaining for PCNA (×200), cytokeratins AE1/AE3 (×200) and EGFr (×400) respectively of DU145 cells after fixation in 10% NBF for 5 min (A), 684 hrs (B) or 12 hrs followed by transfer to 70% ethanol for 672 hrs (C).

In both DU145 and SKOV3 cells, immunostaining for PCNA was undetectable (Figure 1 A, B) after 10% NBF fixation for 684 hrs. In both cell lines, the immunostaining score after 10% NBF fixation for 12 hrs followed by transfer to 70% ethanol was reduced over 50% when compared with 10% NBF fixation for 5 min. Thus transfer to 70% ethanol following 12 hrs of fixation in 10% NBF did not adequately preserve immunorecognition for PCNA e.g., DU145 cells (Figure 2 Panel I).

Evaluation of staining for cytokeratins AE1/AE3 identified a significant reduction (*p*<0.01) in staining after 10% NBF fixation for 684 hrs when compared to 10% NBF fixation for 5 min in both DU145 and SKOV3 cells. In both cell lines immunostaining score after 10% NBF fixation for 12 hrs followed by transfer to 70% ethanol for 672 hrs was significantly higher than 10% NBF fixation for 684 hrs; however, for DU145 cells, staining following 12 hrs of 10% NBF and transfer to 70% ethanol for 672 hrs was less than 50% of immunostaining following 5 min of fixation in 10% NBF (*p*<0.01) (Figure 2 Panel II) but in SKOV3 cells, there was no statistically significant difference between 10% NBF fixation for 5 min compared with 12 hrs of 10% NBF fixation followed by transfer to 70% ethanol.

In DU145 cells, evaluation of cytoplasmic staining for EGFr demonstrated no significant difference when 10% NBF fixation for 684 hrs or 10% NBF fixation for 12 hrs plus 672 hrs in 70% ethanol was compared with 10% NBF fixation for 5 min (Figure 1 A and Figure 2 Panel III). In SKOV3 cells, the immunostaining score for cytoplasmic EGFr was significantly higher after 10% NBF fixation for 12 hrs plus 70% ethanol storage for 672 hrs compared to 10% NBF fixation for 5 min; however there was no statistically significant difference observed in immunostaining score between 10% NBF fixation for 5 min when compared with 10% NBF fixation for 684 hrs. While statistically different, the changes in immunorecognition of the phenotypic cytoplasmic expression of EGFr were modest and might not be experimentally important.

In DU145 cells, the membrane staining for EGFr was statistically different between either 10% NBF fixation for 684 hrs or 10% NBF fixation for 12 hrs followed by transfer in 70% ethanol for 672 hrs when compared to 10% NBF fixation for 5 min (*p*<0.01). In SKOV3 cells, there was no significant difference in immunostaining for membrane EGFr between 10% NBF fixation for 5 min compared with 10% NBF fixation for 684 hrs, however the immunostaining score for membrane EGFr staining of SKOV3 cells fixed in 10% NBF fixation for 12 hrs followed by transfer to 70% ethanol for 672 hrs was statistically higher than for cells fixed for 5 min in 10% NBF (*p*<0.01). The extent of this higher expression of membrane EGFr would likely be experimentally important.

### 4 week fixation of DU145 and SKOV3 cells with and without AR

**Figure 3:**
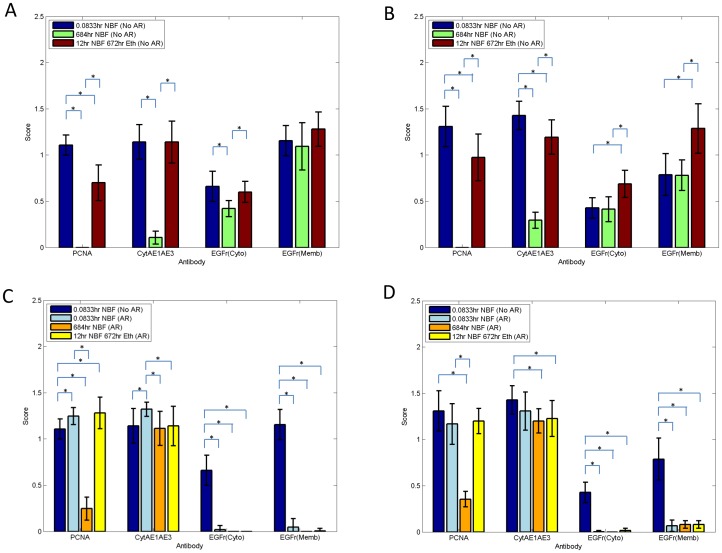
Immunostaining score of DU145 cells (panel A,C) and SKOV3 cells (panel B,D) after fixation in 10% NBF for 5 min or 684 hrs or 12 hrs in 10% NBF plus 672 hrs in 70% ethanol staining for PCNA, cytokeratins AE1/AE3, cytoplasmic or separately membrane staining for EGFr. The top panels show immunostaining scores without AR while bottom panels represent scores with AR. Data from are from three replicate experiments. Error bars are standard deviations (SD) with *n* = 3. * *p* value<0.01.

**Figure 4:**
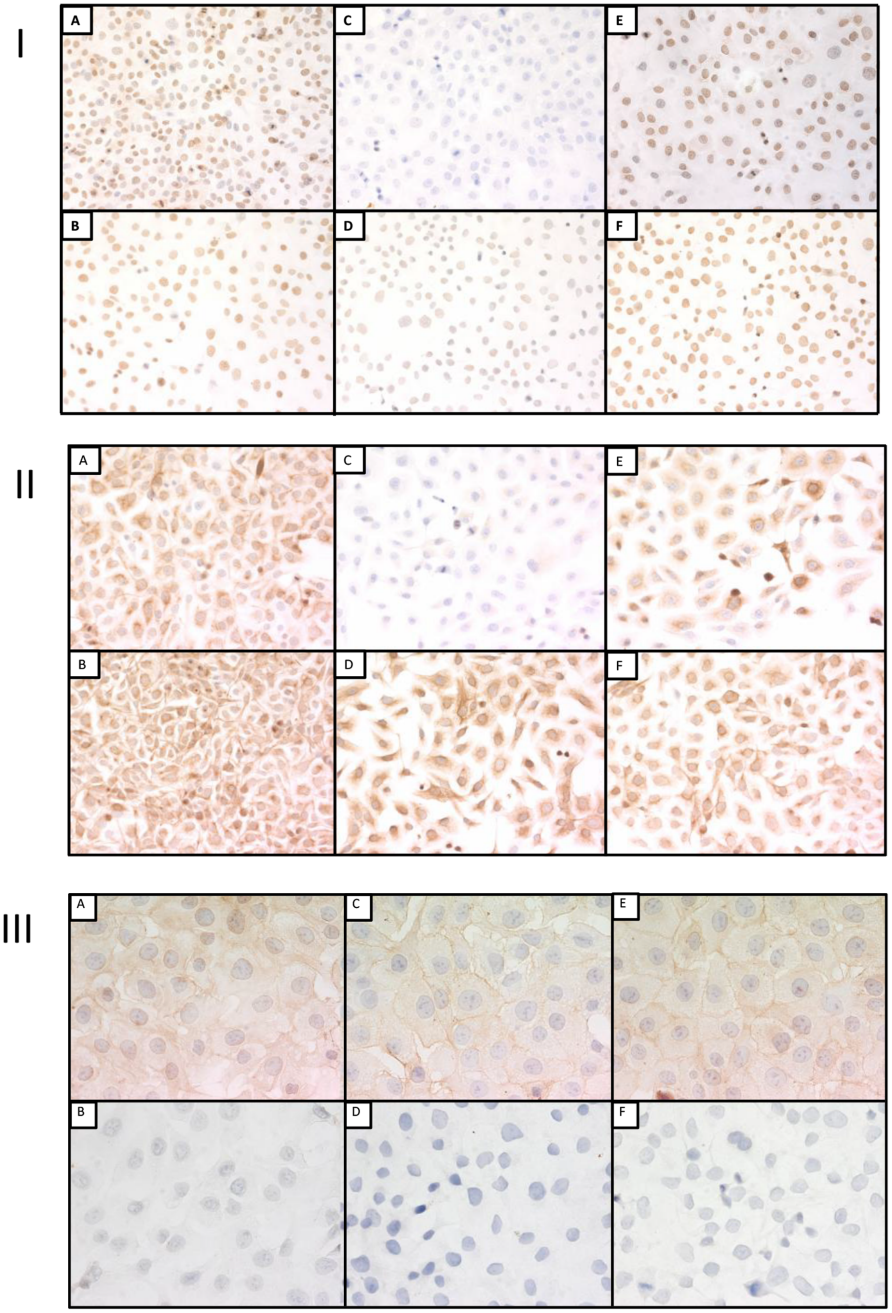
Panels I, II and III represent immunostaining for PCNA (X200), cytokeratins AE1/AE3 (X200) and EGFr (X400) respectively of DU145 cells after fixation in 10% NBF fixation for 5 min, 684 hrs or 12 hrs in 10% NBF followed by transfer to 70% ethanol for 672 hrs. The panels are 10% NBF fixation for 5 min (A), 10% NBF fixation for 5 min with AR (B), 10% NBF fixation for 684 hrs (C), 10% NBF fixation for 684 hrs with AR (D), 10% NBF fixation for 12 hrs plus 672 hrs in 70% ethanol (E) and 10% NBF fixation for 12 hrs plus 672 hrs in 70% ethanol with AR (F).

In experiment 2, staining for PCNA in both cell lines were very similar to experiment 1 i.e., 10% NBF fixation for 684 hrs resulted in a score of approximately zero while fixation in 10% NBF for 12 hrs followed by transfer to 70% ethanol for 672 hrs was higher than 10% NBF fixation for 684 hrs (Figure 3 A, B) but still was significantly lower (*p*<0.01) than 5 min of fixation in 10% NBF e.g., DU145 cells (Figure 4 Panel I).

In both DU145 and SKOV3 cells, immunostaining scores of cells staining for PCNA after AR were significantly higher when compared with respective pairs not exposed to AR (i.e., 5 min −AR vs 5 min +AR; 684 hrs −AR vs 684 hrs +AR or 12 hrs followed by transfer to 70% ethanol for 672 hrs −AR vs 12 hrs followed by transfer to 70% ethanol for 672 hrs +AR) except in SKOV3 cells where no statistical difference was observed in immunostaining scores for PCNA when 10% NBF fixation for 5 min −AR was compared with 10% NBF fixation for 5 min +AR (*p* = 0.09).

Of importance, for PCNA, AR resulted in a small recovery of immunorecognition for specimens fixed in 10% NBF for 684 hrs. In contrast, there was a complete recovery of immunorecognition by AR for specimens fixed for 12 hrs in 10% NBF and transferred to 70% ethanol for 672 hrs.

When immunostaining for cytokeratins AE1/AE3 without AR was evaluated, in both DU145 and SKOV3 cells, 10% NBF fixation for 684 hrs resulted in a large and statistically significant decrease (*p*<0.01) in staining when compared with 10% NBF fixation for 5 min. There were cell line differences observed in evaluation of immunostaining scores for cytokeratins after fixation in 10% NBF for 12 hrs followed by transfer to 70% ethanol for 672 hrs. In DU145 cells, comparing 10% NBF 5 min staining with 10% NBF fixation 12 hrs plus 672 hrs in 70% ethanol was not statistically significant (*p* = 1.0) (Figure 4 Panel II); however, in SKOV3 cells immunostaining score was significantly lower, (*p*<0.01). In both cell lines, exposure to AR resulted in an immunostaining scores for cytokeratins AE1/AE3 similar to those obtained with 10% NBF fixation for 5 min without AR.

In experiment 2, results of immunostaining score for cytoplasmic EGFr were similar to experiment 1. For DU145 cells, like in experiment 1 there were no statistically significant differences in cytoplasmic expression of EGFr between 10% NBF fixation for 5 min and 10% NBF fixation for 12 hrs followed by transfer to 70% ethanol for 672 hrs; however, when fixation for 5 min in 10% NBF and 10% NBF fixation for 684 hrs were compared, cytoplasmic staining for EGFr was significantly but only slightly decreased, (*p*<0.01). In SKOV3 cells, 10% NBF fixation for 12 hrs followed by transfer to 70% ethanol for 672 hrs was significantly higher when compared with either fixation of 10% NBF for 5 min or 10% NBF fixation for 684 hrs. This was unexpected given our hypothesis that all values for immunostaining at other conditions of fixation should be less than that for 5 min of 10% NBF fixation.

In DU145 cells, there was no statistically significant change in immunostaining score for EGFr membrane staining between 10% NBF fixation for 5 min and for 684 hrs (*p* = 0.45) or between 10% NBF fixation for 5 min and 10% NBF fixation for 12 hrs plus 672 hrs in 70% ethanol (*p* = 0.06) (Figure 4 Panel III). In SKOV3 cells, 10% NBF fixation for 12 hrs followed by transfer to 70% ethanol for 672 hrs, was significantly increased, (*p*<0.01), when compared with 10% NBF fixation for 5 min or 10% NBF fixation for 684 hrs. This also was unexpected.

In both cell lines, exposure to AR resulted in essentially a complete loss of both cytoplasmic and membrane staining for EGFr at all experimental treatments and time points.

## Discussion

The fixation of tissues and cells in aldehyde fixatives is known to reduce antigen immunorecognition of some epitopes with increasing time of fixation [5]–[8]. To mitigate this gradual loss of immunorecognition, samples, anecdotally and largely through experience, have been transferred to 70% ethanol after initial fixation in 10% NBF. Our laboratory previously investigated the benefits of this approach by comparing fixation in only 10% NBF with 10% NBF fixation for 12 hrs followed by transfer to 70% ethanol for up to one week. In the prior study, transfer to 70% ethanol was demonstrated to preserve immunorecognition of PCNA, cytokeratins AE1/AE3, Ki67 MIB-1, cytoplasmic and membrane EGFr when compared to fixation in 10% NBF which resulted in almost complete loss of immunorecognition in staining for PCNA, and a significant reduction for cytokeratins AE1/AE3; however, cytoplasmic and membrane phenotypic expression of EGFr was only slightly affected by fixation in 10% NBF [8].

The current study was designed to determine if longer term (i.e., up to 4 weeks) storage in 70% ethanol following 12 hrs of fixation in 10% NBF could similarly prevent the loss on immunorecognition and if antigen retrieval would just be as effective in recovering immunostaining.

The extensive loss of immunorecognition of PCNA and cytokeratins AE1/AE3 was similar upon fixation with 10% NBF for 1 or 4 weeks; however, fixation in 10% NBF for 12 hrs followed by transfer to 70% ethanol for 4 weeks (672 hrs) preserved less than 50% of immunorecognition of PCNA in DU145 and SKOV3 cells in contrast to the almost complete preservation immunorecognition of PCNA after 168 hrs of storage in 70% ethanol. The immunorecognition of cytokeratins AE1/AE3 was more variable; however, based on experiments 1 and 2 it is likely that the immunorecognition of cytokeratins is preserved to a large extent upon storage in 70% ethanol for up to 4 weeks after initial fixation for 12 hrs in 10% NBF.

**Figure 5:**
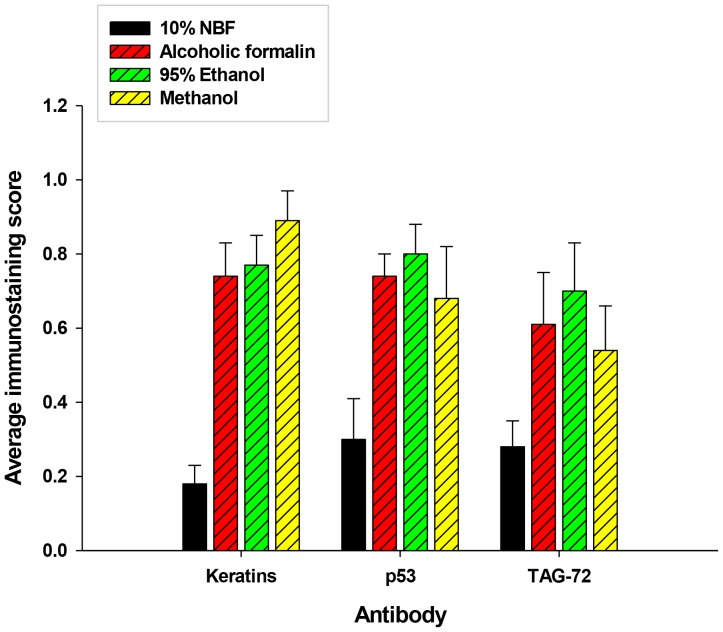
The effects of immunostaining tumor tissue after fixation with alcohol based fixatives compared with 10% NBF. The specimens were in the various fixatives for 18 to 36

As was noted previously, neither cytoplasmic nor membrane expression of EGFr is very sensitive to long term fixation in 10% NBF [8]. The relatively large increase in cytoplasmic and membrane staining of EGFr noted in SKOV3 cells after transfer to 70% ethanol for 4 weeks in experiments 1 and 2 indicated that this method would be problematic in that the effects of on immunorecognition are too variable. The mechanisms of the increase in EGFr expression after 12 hrs of 10% NBF fixation followed by transfer to 70% ethanol in SKOV3 cells are unclear; however, this increase was not noted in DU145 cells which overexpress EGFr. Also, Arnold et al. reported that 95% ethanol, methanol and alcoholic formalin were preferable to other methods of fixation including 10% NBF for immunorecognition of cytokeratins AE1/AE3, p53and TAG-72 when compared to 10% NBF (Figure 5) [5].

The extent to which heat-buffer AR unmasks immunorecognition of formalin fixed epitopes is still not known for many antigens [13] and even less is known of the effects of AR on immunorecognition after short-term exposure to 10% NBF followed by transfer to 70% ethanol. Of note, in DU145 and SKOV3 cells fixed in only 10% NBF, AR inadequately restored immunostaining for PCNA but almost completely restored immunorecognition for cytokeratins, AE1/AE3. This is in agreement with Shi et al. [13] who noted variable recovery of immunorecognition based on the epitope-antibody pair and the type of AR. Of note, just the detection of an antigen does not indicate adequate or consistent recovery of immunorecognition.

In contrast, 10% NBF fixation for 12 hrs followed by transfer to 70% ethanol plus AR resulted in recovery of immunorecognition of PCNA and cytokeratins AE1/AE3; however, unexpectedly, AR resulted in almost complete loss of staining for both cytoplasmic and membrane EGFr regardless of whether the cells were fixed in only 10% NBF or fixed in 10% NBF for 12 hrs followed by transfer to 70% ethanol. Although manufacturer instructions suggest that AR is not required for EGFr immunorecognition (pepsin pretreatment is recommended), this observation emphasizes that heat induced AR is not suitable for some antigens. In this case, two typical methods currently used for AR, pH 6.0 citrate buffer and pH 8.0 EDTA solution actually destroyed the ability to detect EGFr.

Although the term “storage in 70% ethanol” is used because it describes the approach, this terminology does not imply that such storage in 70% ethanol is a passive process with respect to molecular effects (i.e., it is also a fixation process) as is indicated by our prior study [8] and results using AR for cells fixed for 4 weeks in 10% NBF versus cells fixed for 12 hrs in 10% NBF followed by 4 weeks in 70% ethanol. The extent of loss of immunorecognition caused by fixation in 10% NBF varies with the epitopes of the antigen, the monoclonal antibody used to identify the epitope, and the time of fixation. Thus, each epitope – monoclonal antibody pair may be affected differently by fixation in 10% NBF over time, by methods of antigen recovery as well as by transfer to 70% ethanol following initial fixation in 10% NBF. Also, the epitopes of antigens may vary among different cell types which can lead to differences in immunostaining of different cell types in response to fixation which is a limitation of our study. Another limitation is that observations may not apply to all immunodetection by other antigen-antibody pairs in that immunorecognition of EGFr was not as sensitive to the effects of 10% NBF fixation.

This study was performed using a cell model without tissue processing to paraffin. A prior study using a similar cell model found that fixation followed by tissue processing to paraffin combined have to a greater effect on immunorecognition that just fixation or tissue processing alone [7]. Thus, we hypothesize that our observation on loss of immunorecognition will be magnified in tissues when they are processed to paraffin. Verification of this hypothesis is underway.

This study concludes that transfer of cells to 70% ethanol for 4 weeks following 12 hrs of fixation in 10% NBF does not preserve immunorecognition of PCNA and cytokeratins AE1/AE3 as reported for shorter periods of 180 hrs in 70% ethanol [8]. Also, antigen retrieval (AR) did not recover much immunorecognition of these fixation sensitive antigens when cells were fixed in only 10% NBF for 4 weeks, but did recover immunorecognition for those cells that were transferred to 70% ethanol. This suggests that there are beneficial effects on immunorecognition of transfer to 70% ethanol when followed by AR. In contrast, immunorecognition of EGFr is less affected by fixation in only 10% NBF, but if transferred to 70% ethanol for 4 weeks, there is an apparent increase in immunostaining above initial levels and complete loss when exposed to AR. Thus, investigators should be wary of the use of 70% ethanol alone for relatively long periods to maintain immunorecognition.
